# OASI2: a cluster randomised hybrid evaluation of strategies for sustainable implementation of the Obstetric Anal Sphincter Injury Care Bundle in maternity units in Great Britain

**DOI:** 10.1186/s13012-021-01125-z

**Published:** 2021-05-22

**Authors:** Magdalena Jurczuk, Posy Bidwell, Dorian Martinez, Louise Silverton, Jan Van der Meulen, Daniel Wolstenholme, Ranee Thakar, Ipek Gurol-Urganci, Nick Sevdalis

**Affiliations:** 1 Centre for Quality Improvement and Clinical Audit, Royal College of Obstetricians and Gynaecologists, 10-18 Union Street, London, SE1 1SZ UK; 2 Royal College of Midwives, 10-18 Union Street, London, SE1 1SZ UK; 3 Department of Health Services Research and Policy, London School of Hygiene and Tropical Medicine, 15-17 Tavistock Place, London, WC1H 9SH UK; 4 Croydon University Hospitals NHS Trust, 530 London Road, Croydon, CR7 7YE UK; 5 Centre for Implementation Science, Health Service and Population Research Department, King’s College London, De Crespingy Park, London, SE5 8AF UK

**Keywords:** OASI Care Bundle, Obstetric anal sphincter injury, Severe perineal tear, Scale-up, Quality improvement, Implementation

## Abstract

**Background:**

The Obstetric Anal Sphincter Injury (OASI) Care Bundle comprises four primary and secondary prevention practices that target the rising rates of severe perineal tearing during childbirth, which can have severe debilitating consequences for women. The OASI Care Bundle was implemented in 16 maternity units in Britain in the OASI1 project (2017-2018), which demonstrated the care bundle’s effectiveness in reducing OASI rates. In OASI2, the care bundle will be scaled up to 20 additional National Health Service (NHS) maternity units in a hybrid effectiveness-implementation study that will examine the effectiveness of strategies used to introduce, implement and sustain the care bundle.

**Methods:**

OASI2 is a two-arm cluster-randomised control trial (C-RCT) of maternity units in England, Scotland and Wales, with an additional non-randomised study arm. C-RCT arm 1 (peer support, *n* = 10 units) will be supported by ‘buddy’ units to implement the OASI Care Bundle. C-RCT arm 2 (lean implementation, *n* = 10 units) will implement without external support. The additional study arm (sustainability, *n* = 10 units) will include some original OASI1 units to evaluate the care bundle’s sustainability and OASI rates over time, from before OASI1 and through the end of OASI2. Units in all three study arms will receive an Implementation Toolkit with training resources and implementation support. The C-RCT arms will be compared in terms of OASI rate reduction (primary effectiveness outcome) and clinicians’ adoption of the care bundle (primary implementation outcome). Clinical data will be collated from maternity information systems; implementation data will be collected through validated surveys with women and clinicians, supplemented by qualitative methods. Descriptive statistics and regression modelling will be used for analysis. Emergent themes from the qualitative data will be assessed using framework analysis.

**Discussion:**

OASI2 will study the impact of various implementation strategies used to introduce and sustain the OASI Care Bundle, and how these strategies affect the bundle’s clinical effectiveness. The study will generate insights into how to effectively scale-up and sustain uptake and coverage of similar interventions in maternity units. A locally adaptable ‘implementation blueprint’ will be produced to inform development of future guidelines to prevent perineal trauma.

**Trial registration:**

ISRCTN26523605

**Supplementary Information:**

The online version contains supplementary material available at 10.1186/s13012-021-01125-z.

Contributions to the literature

- This randomised hybrid implementation-effectiveness trial aims to determine whether a previously evaluated OASI-reducing care bundle can be implemented with moderate or limited implementation support and still achieve significant clinical effect.
- Simultaneously, this study will continue to work with maternity units that implemented the care bundle in a previous study to identify strategies that successfully support long-term sustainability.
- This study applies several implementation science frameworks and measures and will allow determination of expected mechanisms of implementation. The study will contribute detailed findings regarding what implementation strategies worked or did not work in maternity settings and, importantly, why.

## Background

Care bundles have become a popular approach to improving quality of care in the last decade. The Institute for Healthcare Improvement (IHI) first defined care bundles in 2001 as ‘a small set of evidence-based practices that, when implemented together, will result in significantly better outcomes than when implemented individually’ [1]. The OASI Care Bundle (OASI-CB) was developed in response to a tripling in rates of reported obstetric anal sphincter injury (OASI) in England over a 10-year period [2]. OASI is the collective term for a third- or fourth-degree perineal tears, a severe complication of vaginal childbirth that may have long-term consequences including chronic pain, sexual dysfunction, and urinary and/or anal incontinence [3, 4]. Approximately one in 13 primiparous women with assisted vaginal births and one in 20 primiparous women with unassisted (spontaneous) vaginal births in Great Britain (GB) are reported to experience an OASI [5]. Over half of the women who sustain an OASI have continued morbidities and close to half report an impact on their future birth choices [6]. Furthermore, OASI has significant resource implications for healthcare providers due to ongoing follow-up and litigation [7].

**Fig. 1:**
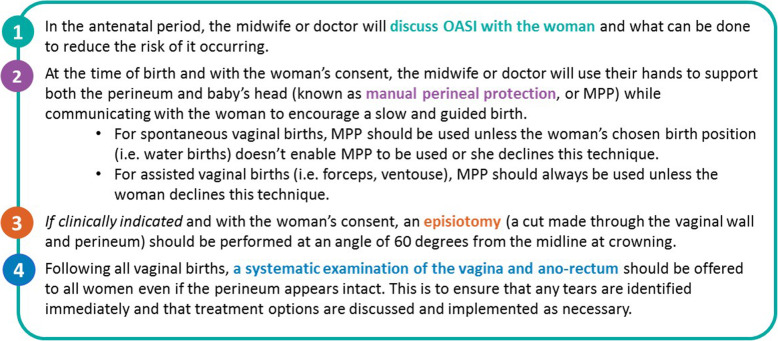
Summary of the OASI-CB’s four components

A multidisciplinary working group of experts from the Royal College of Midwives (RCM), Royal College of Obstetricians and Obstetricians (RCOG) and the London School of Hygiene and Tropical Medicine (LSHTM) developed the OASI-CB—a set of four routine practices in maternity care that support the prevention and early detection of severe perineal tearing (see Fig. 1).

The OASI-CB was implemented and evaluated in a multicentre quality improvement (QI) project (OASI1) with a stepped-wedge design from 2016 to 2018 across 16 NHS maternity units in England, Scotland and Wales [8]. The OASI1 study’s analysis included 55,060 singleton live vaginal births and demonstrated the bundle’s clinical effectiveness: there was a reduction of 20% in the case-mix-adjusted risk of severe perineal injury after the introduction of the OASI Care Bundle (*p* = 0.03), with no effect on caesarean birth or episiotomy rates [9]. Additionally, a process evaluation within OASI1 found that the OASI-CB was acceptable, appropriate and feasible for clinicians and women [10, 11].

A variety of other care bundles have been introduced within maternity care, including ones targeting perinatal depression [12], stillbirth [13], surgical site infection after caesarean birth [14] and postpartum haemorrhage [15]. Despite the rising interest in and use of care bundles, few studies have examined the mechanisms for successful and sustainable implementation at scale. A review published in 2019 confirms that the few studies that report on care bundle implementation processes lack the detail and standardisation necessary to enable replication of findings to support the adoption of other care bundles [16].

Implementation of the OASI-CB in OASI1 relied on continuous, centralised support from an externally funded Project Team, an approach that is not always feasible for scale-up, especially at national scale. This study—OASI2—will compare how different levels of implementation support impact on maternity units’ uptake and utilisation of the care bundle to better understand how to accomplish sustainable, large-scale implementation of the OASI-CB in maternity units across GB. Specifically, OASI2 is a hybrid effectiveness-implementation trial that focuses on how peer-supported facilitation of care bundle implementation compares with unsupported facilitation. The two primary objectives of OASI2 are:

1. To investigate mechanisms and strategies that support the ongoing sustainability of the OASI-CB implementation in a sample of the OASI1 study units
2. To compare effectiveness and implementation outcomes of two scale-up methods (peer support and lean implementation) in units implementing the OASI-CB for the first time.

A secondary objective is to understand women’s perspectives regarding the four elements of the care bundle and their impact on women’s birth experiences.

## Methods/design

### Conceptual frameworks

The study design is underpinned by one determinant framework (defines determinants that act as barriers or enablers to implementation), and one evaluation framework (guides evaluation of implementation) [17]. Application of the determinant framework, Promoting Action on Research Implementation in Health Services (PARIHS) [18], is described in the Intervention section, and application of the Implementation Outcomes evaluation framework [19] is described in the Evaluation section.

### Design overview

OASI2 is a two-arm cluster randomised control trial (C-RCT) with an additional, parallel study arm of purposively invited units. The additional study arm is referred to as the ‘sustainability’ arm as it constitutes units that participated in the original OASI1 study and their efforts are now focused on sustaining the care bundle over time. The units in the sustainability arm will continue to be centrally supported by the Project Team under the ‘expert outreach’ facilitation model.

In all three arms, each unit’s senior leadership will designate one obstetrician and one midwife to take on the role of OASI QI Lead. The role of the OASI QI Leads is to carry out and adapt the recommended implementation strategies for the local context. The Project Team will provide all unit leads with an Implementation Toolkit. The toolkit includes:

- A clinical manual for midwives and obstetricians that describes how to apply the four components of the OASI-CB
- Access to an eLearning programme that includes a clinical module to support skills training
- An implementation guidebook that serves as a practical blueprint for care bundle roll-out by outlining how and when to facilitate six key implementation strategies
- Promotional materials such as posters and other visual reminders of the OASI-CB

These resources were selected and developed based on experiences and lessons learned from the OASI1 study.

The units that make up the C-RCT will be randomised to one of two arms and will implement the OASI-CB under different facilitation models:

1. Units randomised to C-RCT arm 1 will implement the OASI-CB with ‘peer support’ from a nearby maternity unit of the study’s sustainability arm.
2. Units randomised to C-RCT arm 2 will implement the OASI-CB without peer support. The resources provided to this group of units are limited to the contents of the Implementation Toolkit. This facilitation model is referred to as ‘lean’ implementation.

**Fig. 2:**
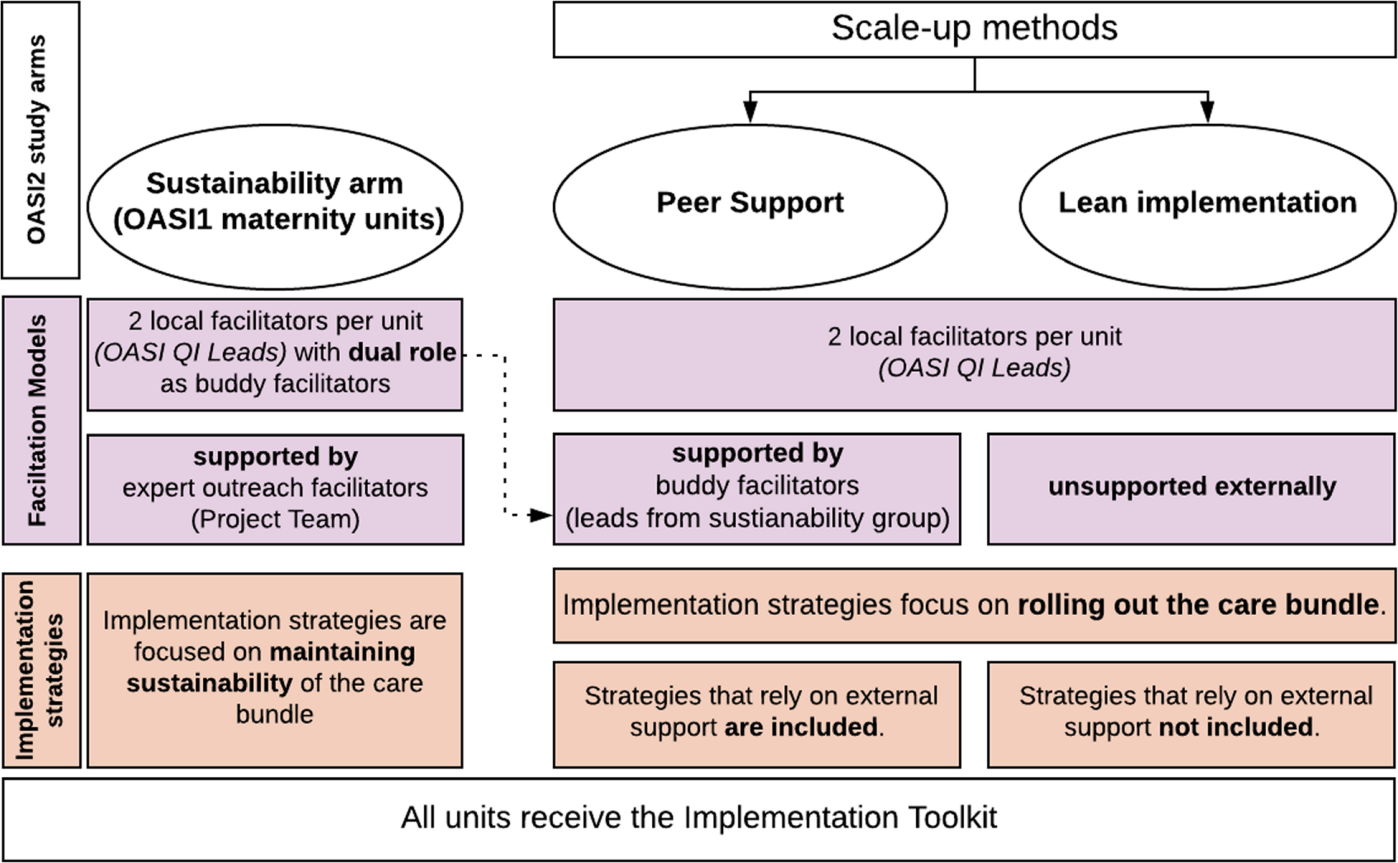
Overview of OASI2 study design

Figure 2 summarises the study arms.

### Study participants

Thirty NHS maternity units across GB will make up the three study arms (the sustainability arm, and the two C-RCT arms, peer support and lean implementation) with ten units in each (see power calculations in the evaluation section).

Inclusion criteria for units in the sustainability arm are:

- Participation in the OASI1 study
- Attendance and active participation at a co-design event after OASI1
- A letter of commitment indicating willingness to sustain the OASI-CB at the organisational level, continue extracting requested administrative data, and facilitate protected time for unit leads to support OASI-CB implementation in neighbouring units
- Written confirmation of interest in response to a letter from the Project Team providing additional details about responsibilities of study participants.

Selection of the units for the C-RCT arms began with a call for expressions of interest from the clinical directors and heads of midwifery of all NHS maternity units across England, Scotland and Wales. Seventy units from fifty-six NHS Trusts (England) or Boards (Scotland and Wales) expressed interest to participate. Units from the same trust/board as a sustainability unit and units that participated in the pilot study prior to OASI1 were excluded.

The remaining sixty-four units were divided into six groups based on their geographical proximity to at least one sustainability arm unit. Senior staff from sustainability arm units were contacted to longlist three units from their regional groupings that they are able to work with. From each group of three units, the Project Team shortlisted two units that are most similar to each other in terms of unit type (obstetric unit (OU), alongside midwifery unit (AMU) or freestanding midwifery unit (FMU)) and average number of births per year. Blocking of the candidate units based on the extraneous factor of sustainability unit preference circumvents the potential of pairing up two units that are unable to work together, which would compromise the peer support model evaluated in the trial.

**Fig. 3:**
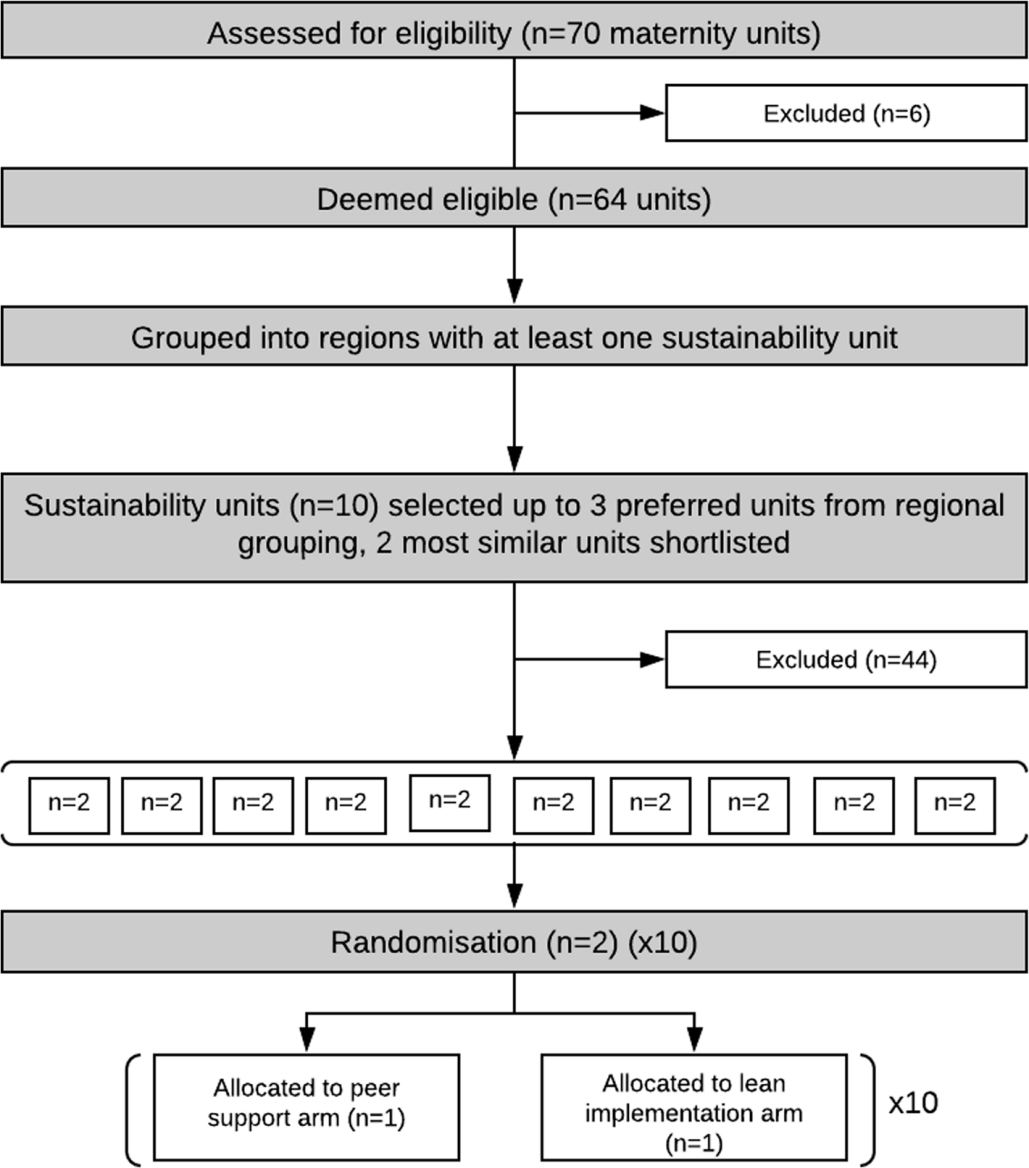
CONSORT flow diagram for selection of C-RCT units

Once the research and development (R&D) departments from all 20 units have confirmed participation and completed local set-up processes, within each of the ten “pairs” of units, units will be randomised into either peer support or lean implementation arms using a random number generator by an independent academic researcher (not associated with institutions linked to the Project Team). Allocation will be concealed from all participating units until the launch of the study to prevent premature contact between paired sustainability arm units and peer support units. The selection and randomisation process is summarised in Fig. 3.

Within each participating maternity unit, there are three groups of participants: the two OASI QI Leads facilitating implementation in each unit, the unit clinicians (midwives and obstetricians), and women who are eligible to receive the care bundle.

### Interventions

#### Implementation mapping and application of the PARIHS framework

Implementation mapping is a practical tool for planning the implementation of evidence-based interventions [20]. Implementation mapping was used to draw on the lessons learned from OASI1 and apply conceptual frameworks to define determinants of implementation success and conceptualise implementation outcomes (see Table 1).

**Table 1:** Implementation mapping applied in OASI2

Implementation mapping steps	Application in OASI2
1. Conduct a needs and assets assessment and identify adopters and implementers	• Review of implementation process and lessons learned in OASI1 • Distinguish between roles of senior staff, implementation facilitators and clinicians
2. Identify adoption and implementation outcomes, performance objectives, and determinants; create matrices of change	• Establish roles, objectives and outcomes for senior staff, implementation facilitators, clinicians, and women receiving the care bundle
3. Choose theoretical methods; select or create implementation strategies	• Selection of the PARIHS framework, implementation outcomes framework • Theory of Change (see Additional file 1) and Logic model development (Fig. 4)
4. Produce implementation protocols and materials	• Tailoring of ERIC strategies across study arms (see Table 2) • Co-design of the Implementation Toolkit with reference to clinicians’ and women’s experiences in OASI1, incorporating results from Shared Learning Day and PPI collaboration
5. Evaluate implementation outcomes	• Plan evaluation of clinical outcomes • Plan evaluation of implementation outcomes

**Fig. 4:**
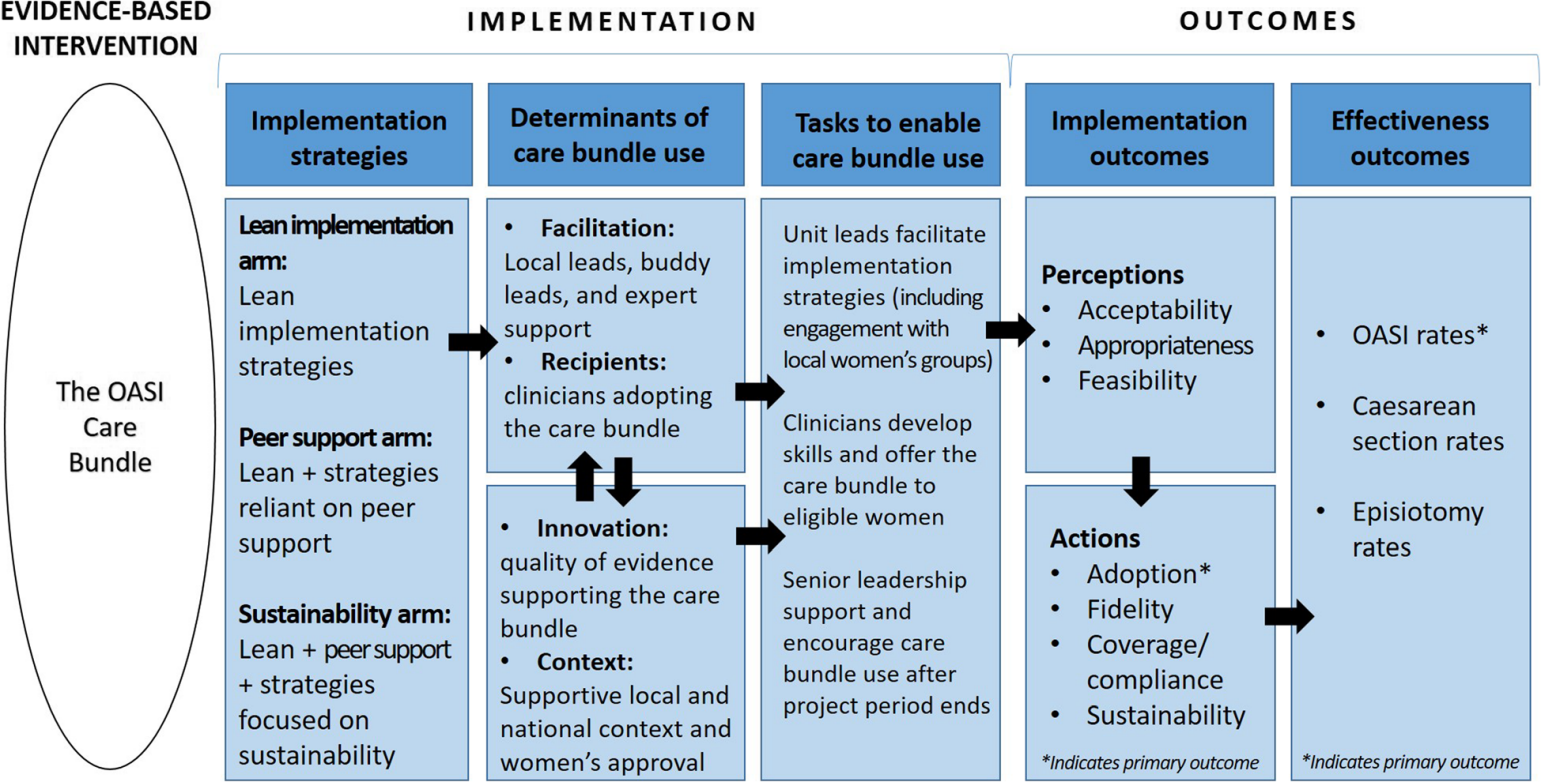
OASI2 logic model developed using implementation mapping

The OASI2 logic model developed as a result of implementation mapping is shown in Fig. 4.

The logic model takes into account the determinants of implementation success as defined by the PARIHS framework, which defines four core ‘determinants’ of implementation success: innovation, context, recipients, and facilitation [18]. ‘Facilitation’ refers to the OASI QI Leads leading implementation and acting as facilitators. ‘Recipients’ are the clinicians ‘receiving’ the implementation and adopting the care bundle into their routine practice. Facilitation and recipients are therefore internal determinants of implementation success. Conversely, ‘innovation’ and ‘context’ are external determinants that may impact the OASI QI Leads and clinicians. Innovation refers to the acceptability and evidence base supporting the four components and the OASI-CB as a whole; depending on the strength of this evidence, this can have a positive or negative impact on OASI QI Leads, clinicians, and ultimately, the implementation. The favourability of the context will further impact implementation. At the national level, this depends on the opinion of professional clinical leadership and support of women’s advocacy groups; a favourable local context includes existing capacity for quality improvement and senior buy-in early in the implementation period. Context also takes into account the approval and support of the care bundle’s target population; women who are eligible to receive the care bundle and women who have received it.

The PARIHS framework considers facilitation to be the “active ingredient” of implementation, acknowledging that facilitator(s) of change in clinical practice may be internal or external and focus on enabling others to make changes [21]. Each of the three study arms are defined by a set of implementation strategies that are influenced by the facilitators involved in the implementation:

- The lean units will rely on their local OASI QI Leads;
- The peer support units have their local leads + the support of experienced buddy leads from nearby units; and
- Sustainability units have their local leads + centralised support from the Project Team.

Implementation strategies for each study arm were selected from the compilation of strategies defined by the Expert Recommendations for Implementation Change (ERIC) project [22] based on scalability and enablers identified in the OASI1 project. Table 2 details how each set of discrete implementation strategies will be operationalised across the three study arms. These sets of strategies are summarised in the sections below, for each study arm.

**Table 2:** Implementation strategies operationalised across three study arms

Study timeline	Operationalised strategy in OASI2	Relevant ERIC strategies	Study arm		
			Lean	Peer support	Sustain-ability
*Set-up*	Heads of midwifery/clinical directors select one obstetrician and one midwife to take on the OASI QI Lead role (informed by a role description)	Identify and prepare champions; facilitation; clinical supervision	X	X	X
	OASI QI Leads get 1 day/month of **protected time** to dedicate to OASI-CB sustainability in own unit and to support buddy unit	Fund & contract for the clinical innovation			X
*Launch*	OASI QI Leads prepared for external facilitator (‘buddy’) role during virtual **skills development days** organised by clinical and implementation experts (Project Team)	Recruit, designate and train for leadership			X
	OASI QI Leads receive the **Implementation Toolkit**	Develop an implementation blueprint; use educational materials	X	X	X
	OASI QI Leads paired up with and introduced to their ‘buddies’	Create a learning collaborative		X	
*Throughout the study period*	OASI QI Leads engage with their ‘buddies’ on a monthly basis (minimum) to seek implementation guidance	Provide local technical assistance; shadow other experts		X	
	OASI QI Leads to receive **centralised support** from clinical and implementation experts (Project Team) via monthly advisory meetings	Centralise technical assistance; use an implementation advisor; provide ongoing consultation; promote network weaving; organize clinician implementation team meetings			X
	Monthly contact between Project Team and heads of midwifery/ clinical directors to encourage senior buy-in from key members of staff within unit i.e. Labour ward lead, Band 7 labour ward co-ordinators, Patient Safety Leads, QI team etc. and to introduce the OASI-CB into local guidelines and mandatory training / induction packages for new staff (obstetricians and midwives)	Involve executive boards; mandate change/ create or change credentialing and/or licensure standards			X

*OASI* obstetric anal sphincter injury, *QI* quality improvement, *OASI-CB* OASI Care Bundle, *ERIC* Expert Recommendations for Implementing Change

#### Sustainability arm implementation strategies

In this arm, strategies focus on solidifying the OASI-CB’s presence by involving and sustaining buy-in from executive boards in order to incorporate it into local guidelines and training packages. The aim is to address organisational barriers and enablers that were identified during OASI1 to assure the care bundle’s long-term sustainability.

OASI QI Leads in this arm have a dual role: in addition to local facilitation to ensure sustainability within their own units, they act as ‘buddy’ facilitators to nearby units in the peer support study arm (C-RCT arm 1). As buddy facilitators, they are expected to contact leads from their buddy units at least once per month and draw on their own experiences to support and advise on effective operationalisation of implementation strategies. They will receive the OASI-CB Implementation Toolkit and continue to receive centralised support from the Project Team as in OASI1. Centralised support from the Project Team is delivered to this study arm through four key strategies:

1. A live, virtual skills development session with clinical and implementation experts
2. A dedicated support day from the Project Team’s clinical leads approximately 2 months after the virtual skills development session to provide additional skills development support
3. One day per month of protected time during the implementation period to be dedicated to OASI QI Lead responsibilities
4. Monthly catch-up calls with OASI QI Leads to follow-up on progress of sustainability efforts

#### Peer support (C-RCT arm 1) implementation strategies

The ten units randomised to this study arm are paired with units from the sustainability arm. The OASI QI Leads in this arm will receive the Implementation Toolkit and benefit further from continuous external support from the sustainability arm, their buddy facilitators. OASI QI Leads in the peer support arm will maintain monthly contact with their buddy facilitators. Continuous peer support is the defining feature of this scale-up method that distinguishes it from lean implementation (see below).

#### Lean implementation (C-RT arm 2) implementation strategies

Units in this arm will be testing the most ‘hands-off’ scale-up method for care bundle implementation. As is standard across the three arms, the two OASI QI Leads will receive the Implementation Toolkit which is designed to guide and support implementation efforts. As most improvement interventions within the NHS do not provide external facilitation or implementation support, this study arm is most similar to current practice and therefore most akin to a control group. Provision of the Implementation Toolkit replicates what sometimes (though not systematically or as specifically) is offered as implementation support [23] or QI support [24] within the NHS.

### Evaluation of OASI2

#### Evaluation framework and study outcomes

**Fig. 5:**
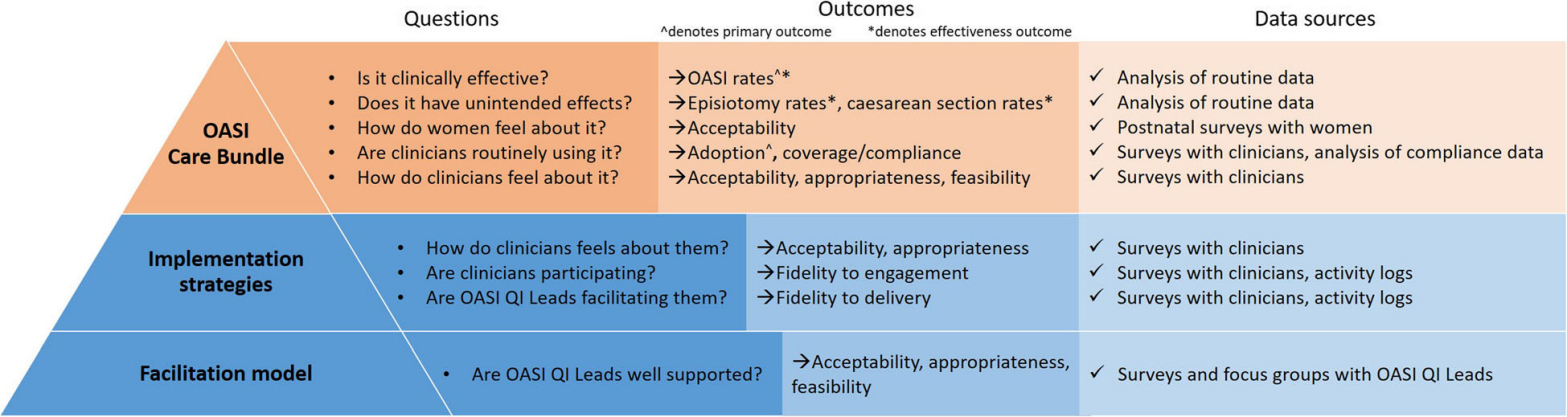
Three-level ‘iceberg model’ with study outcomes and data sources

Clinical and implementation effectiveness data will be collected across all three study arms using mixed methods. All data sources and study outcomes are outlined in Fig. 5, which adopts an ‘iceberg model’ [25] to illustrate the three different levels of perspectives evaluated: the OASI-CB, implementation strategies and facilitation model. Clinical effectiveness outcomes are focused on the OASI-CB, the visible ‘tip of the iceberg,’ while implementation outcomes span all the three levels, elucidating the role and impact of implementation strategies and the facilitation model (peer supported vs. lean implementation).

The Proctor et al. implementation outcome taxonomy [19] guides our evaluation of several implementation outcomes across the three levels shown in Fig. 5. The implementation outcomes are either perception-based or action-based. Acceptability (Is it agreeable, palatable, satisfactory?), appropriateness (Is it compatible?) and feasibility (Is there capacity to do this?) are evaluated based on how an evidence-based intervention (EBI) and/or its implementation are *perceived*. Adoption of the EBI, fidelity (Is the EBI/its implementation carried out as intended?), the EBI’s coverage of the target population and sustainability are evaluated based on *actions taken* or behaviours changed.

The facilitation model is assessed based on how facilitators perceive its acceptability and appropriateness and feasibility.

Implementation strategies are assessed based on whether facilitators deliver the strategies as intended, whether clinicians engage or participate in the strategies as expected (fidelity to the delivery of and engagement with the strategies), and how clinicians perceive their acceptability and appropriateness.

The OASI-CB is assessed in terms of how clinicians perceive its acceptability, appropriateness and feasibility, and whether clinicians adopt the care bundle with fidelity (as intended) into their routine practice and offer it at high coverage. The primary implementation outcome (adoption) and clinical effectiveness outcome (OASI rate) are both measured at this level.

#### Study hypotheses

There are two hypotheses, one related to the primary clinical effectiveness outcome (OASI rates) and one to the primary implementation outcome (OASI-CB adoption):

1. The OASI rate will decrease in all study arms following the implementation of OASI-CB. There will be a significant difference in reduction of OASI rates between units in the peer support and lean implementation arms, such that the reduction in OASI rates will be higher in the peer support arm as compared with the reduction in OASI rates in the lean implementation arm.
2. There will be a significant difference in care bundle adoption between units in the peer support and lean implementation arms, such that the levels of adoption will be higher in the peer support arm.

#### Sample size and power

The sample size calculation aimed to identify the minimum number of clusters (maternity units) required to provide a study power of at least 0.80 with a statistical significance level of 0.05 to detect a 0.5% difference in OASI rates between the peer support and lean implementation arms following the implementation of OASI-CB.

The OASI rate is 3.5% according to the National Maternity and Perinatal Audit (NMPA) [5]. The units in the peer-support arm are expected to achieve a similar rate of reduction in OASI rates as the relative reduction of 20% observed in OASI1, or in absolute terms a reduction in OASI rates from 3.5% to 2.8%. We assume that the relative reduction in the lean implementation arm will be only 5%, or in absolute terms a reduction in OASI rates from 3.5% to 3.3%.

Using the Shiny CRT Calculator [26] by Hemming et al. and a trial design that includes baseline measures, a cross-sectional sampling, an exchangeable correlation structure, a cluster size of 3600 (average maternity unit size according to NMPA), allowance for a varying cluster sizes (coefficient of variation 0.4 according to the NMPA), and an intraclass correlation of 0.007 (according to OASI1), the minimum number of clusters would detect the assumed difference in OASI after implementation of the OASI-CB is ten; therefore, the study will have ten units in each trial arm.

### Clinical effectiveness evaluation

#### Data collection and management

The clinical effectiveness of the OASI-CB is evaluated through its impact on perineal outcomes in women across all participating units. As OASI is an acute pregnancy outcome, women may receive the four elements of the bundle as they become eligible, dependent on whether the attending clinician (midwife or obstetrician) chooses to, or is trained to use the care bundle. Women are not randomised to receive the care bundle. All women who have a vaginal birth (spontaneous or assisted) are eligible to receive the OASI-CB, unless they do not consent to one of the elements of the care bundle or are in a birthing position that makes it unfeasible to implement all elements of the care bundle (e.g. water birth).

Individual woman-level data will be extracted from local electronic Maternity Information Systems (MIS) for all participating units in England, Scotland and Wales. The MIS data extract will include demographic and clinical information related to primary and secondary clinical outcomes, as well as a small set of variables required for cohort derivation and risk adjustment. Data will not be patient identifiable. The full data specification can be found in Additional file 2.

Baseline data extracts will be requested from the 20 randomised units covering 1 year prior to the start of the OASI2 implementation period. For the ten units in the sustainability arm, baseline data extracts will cover the time period between end of the OASI1 study and start of OASI2. All subsequent data extracts during the implementation period will be requested quarterly to enable the Project Team to monitor the quality of the data extracts and to resolve any data-related queries early on. During the implementation period, clinicians will be asked to record their use of (or compliance with) the OASI-CB for every vaginal birth they attend. At minimum, this will be a single yes/no question: ‘were all four components of the OASI-CB applied to this birth?’ Where possible, a field will be added to the local MIS to facilitate collection of these data.

Data from each unit will be cleaned and re-coded to ensure consistent definitions for all variables. Data quality will be assessed by checking data completeness, plausible distributions and internal consistency. Multiple imputation methods (e.g. statistical coefficients obtained from ten imputed data sets pooled using Rubin’s rules [26]) will be used to deal with missing data, if possible, following an assessment of the extent and patterns of missing data. Plausible distribution checks include assessing whether the distributions calculated from non-missing data is within a clinically possible and acceptable range (e.g. OASI rates less than 15%). Internal consistency checks include assessing agreement of data that might be present in more than one data field (i.e. repair of a tear would only be recorded in women who had an OASI). Any implausible distributions or high levels of internal inconsistency would suggest data extraction errors or systematic errors in coding, which would need to be discussed with the unit’s MIS team, and a new revised extract will be requested if required.

#### Data analysis

Routine data extracted from the MIS will be first analysed descriptively, with OASI rates, caesarean birth rates, episiotomy rates and OASI-CB use calculated for all eligible women pre and post implementation and by study arms. Because units in the sustainability arm have already implemented the OASI-CB as part of OASI1, effectiveness outcomes in these units will be analysed differently than in the two C-RCT arms.

For the C-RCT arms, multilevel logistic regression and estimation of adjusted odds ratios (aOR) will be used to assess the impact of the OASI-CB and the relative differences in the odds of OASI in the two C-RCT arms. The regression model will include a random effect to account for clustering at unit level, secular trends and individual case-mix factors (e.g. maternal age, ethnicity, BMI, parity, mode of birth and birthweight).

For the sustainability arm, OASI rates will be analysed longitudinally from the baseline period of OASI1 to end of OASI2, to ascertain the trends and the rate of change by various implementation strategies. The four specific time periods will include baseline (pre-OASI1), OASI1 implementation, post-OASI1 with no external support and OASI2. We will use multilevel logistic regression with random effects and case-mix adjustment as in the C-RCT arms, to estimate adjusted odds ratios (aOR) for each time period and to test the significance of the relative impact of OASI1 implementation as compared with OASI2 implementation to assess the effectiveness of additional “sustainability” strategies.

For all study arms, subgroup analyses of the effect of the care bundle on OASI rates will be carried out according to mode of birth (spontaneous or assisted) and parity. The Wald test will be used to test for significance of interaction terms.

The analysis will be done following intention-to-treat principle, with births analysed according to whether they took place during the baseline or implementation periods, irrespective of whether or not all aspects of the care bundle could be implemented.

### Implementation evaluation

**Fig. 6:**
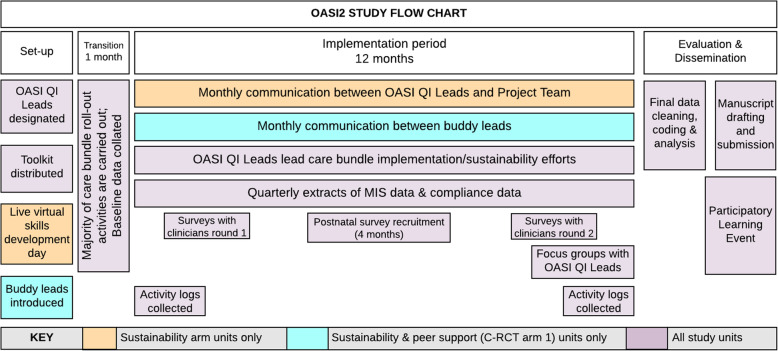
OASI2 study timeline

Four surveys will be used to collect unit-level data: a contextual assessment survey, an Implementation Process Survey for OASI QI Leads, a clinicians’ survey, and a postnatal survey for women. Qualitative methods in the form of activity logs and focus group discussions will also be used. Timing of data collection activities are outlined in Fig. 6.

#### Contextual survey

A contextual survey will complement subsequent analyses and evaluations of OASI-CB implementation at the unit and study arm levels to determine how maternity unit context impacts care bundle implementation. The survey will assess factors identified as significant to implementation OASI1 (i.e. number of staff, number of students, level of research activity) as well as the impact of the COVID-19 pandemic. Each unit’s maternity governance team will complete this survey, administered online, at project start.

#### Implementation process survey with OASI QI Leads

OASI QI Leads will be surveyed at two points during the study: during the first and last three months of the implementation period. These surveys focus on the facilitation model and will measure perceptions of the expert outreach support received by the sustainability arm units, the peer support model or the lean implementation model. These surveys include the previously developed acceptability of intervention measure (AIM), intervention appropriateness measure (IAM) and the feasibility of intervention measure (FIM) [27]. Both OASI QI Leads for all 30 participating units will be invited to complete the survey.

#### Clinicians’ survey

Midwives and obstetricians from participating units will be invited to complete a survey in the first and last three months of the implementation period that will measure:

- Adoption of the OASI-CB
- Perceptions of the OASI-CB
- Perceptions of the implementation strategies
- Participation in implementation strategies

The AIM, IAM and FIM are used as in the survey with OASI QI Leads. Adoption is measured using the Normalization Measurement Development (NoMAD) Tool [28], developed in line with constructs of the Normalization Process Theory, which focuses on the work done at an individual level by adopters of new practices. All midwives and obstetricians will be invited to participate by way of a census method.

#### Women’s postnatal surveys

Women who give birth in participating units will be surveyed about their experiences with the OASI-CB. Survey questions have been extensively reviewed by patient and public involvement (PPI) groups for sensitivity. Over a 4-month period, all women who had a live vaginal birth will be invited to participate in the survey by way of a census method. An attending clinician will approach eligible women at an appropriate time after birth and before discharge from the hospital to explain the survey and provide a participant information sheet and consent form. Participants will receive a link to the online survey via e-mail 2 weeks after the birth of their baby and up to three reminders thereafter if the survey has not yet been completed. Assuming a 30% response rate [29, 30], an estimated 8000 surveys will be completed, which is adequate for descriptive analyses (no comparators for these survey data).

#### Survey data analysis

All survey data will be aggregated for each assessed implementation outcome. For previously validated scales (e.g. AIM, IAM, FIM, NoMAD) internal consistency will be first calculated (Cronbach’s alpha coefficients). Assuming acceptable reliability (Cronbach’s alpha of 0.70 or higher), item ratings will be aggregated into scale scores.

Multivariate regression analyses with clinicians’ survey data will explore the relationship between favourable care bundle perceptions and high care bundle adoption (outcomes) and implementation strategies used and facilitation model assigned to the study arm (predictors). Analyses will also explore the impact of contextual factors (size and type of unit, prevalence of students, staff turnover rates).

Results from the OASI QI Leads’ and clinicians’ surveys from the two time points will be compared within units to assess rating/score variation over time.

Fidelity assessment will be carried out as follows: OASI QI Leads’ survey data for each outcome will be compared with clinicians’ awareness of, participation in and perceptions of the discrete implementation strategies used in unit from the clinicians’ survey. Clinicians’ awareness of strategies in their unit serves as a proxy indicator of OASI QI Leads’ fidelity to the prescribed implementation strategies. Clinicians’ participation in and perceptions of the strategies will be taken as indicators of the clinicians’ fidelity to engagement with the strategies.

Women’s postnatal survey data will be analysed descriptively by each of the OASI-CB components and will be compared between units within study arms. No specific hypotheses will be tested in relation to this dataset.

#### Implementation activity logs

To gain insight into the units’ implementation processes, OASI QI Leads will keep a log of their implementation efforts and share it with the Project Team at two time points: after the 1-month transition period and after the 12-month implementation period. Twenty logs (one from each unit) will be received by the Project Team at the two time points. As a quality and completeness assurance measure, a member of the Project Team will schedule a telephone call with each unit (with both unit leads, if possible) to follow up on the submitted logs and to fill in any missing information.

The Project Team will have monthly contact with OASI QI Leads from the sustainability arm, which will be guided by the activity logs; leads will therefore be asked to share activity logs on a monthly basis.

Each planned and reported activity will be matched to one or more of the 73 discrete implementation strategies compiled by the ERIC Project, as described in Table 2. Content analysis will be used to code any obstacles reported by OASI QI Leads. The matched strategies, coded obstacles and additional information collected in the logs such as number of participants and amount of time spent will be entered in a data file. The information will be identifiable by unit and study arm. After the second round of activity logs are received and entered into the file, the data will be analysed descriptively to identify any patterns between implementation strategies and the facilitation model.

#### Focus groups with OASI QI Leads

Focus group discussions (FGDs) will be conducted with OASI QI Leads to further understand the scalability of the different facilitation models and the operationalisation of the different implementation strategies. The discussion will be guided by the study’s underpinning conceptual frameworks.

Each FGD will have six to eight participants, comprising an even distribution of obstetric and midwifery leads. The FGDs will be conducted virtually and will take approximately 60 min. FGDs will be recorded and transcribed verbatim and field notes summarised. All transcripts will be anonymised and de-identified.

There will be three sets of FGDs specific to each of the three facilitator roles. Each FGD will only have leads from the same study arm. Leads will be strategically assigned/distributed as to not be grouped with their co-lead in a focus group. The number of FGDs conducted depends on the repetition of emergent themes (data saturation) [31].

A deductive approach will be taken to the framework analysis method through the use of PARIHS framework and Normalisation Process Theory constructs to guide data collection and its subsequent coding. The framework analysis method is well-suited for comparing and contrasting data across themes, which is appropriate as we seek to compare the two facilitation models of peer implementation and lean implementation [32].

### Synthesis of clinical effectiveness and implementation data

If compliance (clinicians’ use of the OASI-CB) and survey data are of sufficient completeness and quality, this unit level data will be added to multivariate regression models with effectiveness data to identify and estimate any relationship between OASI rates and levels of OASI-CB adoption as well as any secondary implementation outcomes. For the two C-RCT arms, the regression analysis will assess the impact of care bundle adoption (based on NoMAD score from clinicians’ survey) on relative differences of odds of OASI. In the sustainability arm, the regression analysis will assess the impact of additional ‘sustainability strategies’ on the adoption of the care bundle and on odds of OASI.

### Women’s and stakeholder involvement

Women’s involvement is central to the development of this study. Women's insights on both the implementation and evaluation of OASI2 have been prioritised and incorporated. As the study progresses, women’s involvement will continue to guide study activities, including how to support participating maternity units to effectively engage with women and to embed this engagement in local practice.

As part of the study’s governance of OASI2, an Independent Advisory Group (IAG) provides the OASI2 project team with expert, independent advice and will provide recommendations on implementation and evaluation. The OASI2 IAG consists of clinicians representing obstetrics, gynaecology and midwifery; methodological and clinical leads; implementation and mobilisation science experts; women’s representatives; and representatives from two women’s stakeholder organisations, MASIC (Mothers with Anal Sphincter Injuries in Childbirth) and the Birth Trauma Association (BTA). The IAG and project team meet every 6 months, at a minimum.

## Discussion

OASI2 is a pragmatic, theory-driven hybrid effectiveness-implementation study that will evaluate the implementation of a care bundle in UK NHS maternity units by identifying and elucidating the role of specific stakeholders and other factors that act as determinants of implementation success in addition to using standardised taxonomy in the reporting and study of implementation strategies. A pragmatic approach is taken in the study design as well as the development of implementation materials and the monitoring and evaluation plan. The two implementation support methods to be compared were selected as feasible options for national scale-up. The Implementation Toolkit was developed by experienced clinicians and aims to assist implementation by passing on lessons learned in a way that can be both operationalised and localised. Study findings will be shared widely through peer-reviewed publications, evidence briefs, and media/social media channels. Beyond OASI2, through triangulation of data and theory, this study aspires to create a more thorough understanding of barriers and enablers to the sustainable, large-scale implementation of care bundles in maternity care.

## Supplementary Information

**Additional file 1.** Theory of Change for OASI2

**Additional file 2.** Summary of data specification for the OASI2 project

## Data Availability

Not applicable

## Abbreviations

IHI: Institute for Healthcare Improvement
OASI: Obstetric anal sphincter injury
RCM: Royal College of Midwives
RCOG: Royal College of Obstetricians and Gynaecologists
LSHTM: London School of Hygiene and Tropical Medicine
QI: Quality Improvement
PARIHS: Promoting Action Research for Improving Health Services
EBI: Evidence-based intervention
C-RCT: Cluster randomised control trial
ERIC: Expert Recommendations for Implementing Change
MIS: Maternity Information System
AIM: Acceptability of intervention measure
IAM: Intervention appropriateness measure
FIM: Feasibility of intervention measure
NoMAD: Normalization Measurement Development
aOR: Adjusted odds ratio

## References

[1] Resar R, Griffin FA, Haraden C, Nolan TW. Using Care Bundles to Improve Health Care Quality. IHI Innovation Series white paper. Cambridge, Massachusetts: Institute for Healthcare Improvement; 2012. (Available on www.IHI.org).

[2] Gurol-Urganci I, Cromwell DA, Edozien LC, Mahmood TA, Adams EJ, Richmond DH, Templeton A, van der Meulen J (2013). Third- and fourth-degree perineal tears among primiparous women in England between 2000 and 2012: time trends and risk factors. BJOG An Int J Obstet Gynaecol.

[3] Taithongchai A, Veiga SI, Sultan AH, Thakar R (2020). The consequences of undiagnosed obstetric anal sphincter injuries (OASIS) following vaginal delivery. Int Urogynecol J.

[4] Fornell EU, Matthiesen L, Sjödahl R, Berg G (2005). Obstetric anal sphincter injury ten years after: subjective and objective long term effects. BJOG An Int J Obstet Gynaecol.

[5] NMPA Project Team. National Maternity and Perinatal Audit: Clinical Report 2019. Based on births in NHS maternity services between 1 April 2016 and 31 March 2017. London: RCOG; 2019.

[6] Evans E, Falivene C, Briffa K, Thompson J, Henry A (2020). What is the total impact of an obstetric anal sphincter injury? An Australian retrospective study. Int Urogynecol J.

[7] Mellgren A, Jensen LL, Zetterström JP, Wong WD, Hofmeister JH, Lowry AC. Long-term cost of fecal incontinence secondary to obstetric injuries. Diseases of the colon & rectum. 1999;42(7):857–65. 10.1007/BF02237089 10411431

[8] Bidwell P, Thakar R, Sevdalis N, Silverton L, Novis V, Hellyer A (2018). A multi-centre quality improvement project to reduce the incidence of obstetric anal sphincter injury (OASI): study protocol. BMC Pregnancy Childbirth.

[9] Gurol-Urganci I, Bidwell P, Sevdalis N, Silverton L, Novis V, Freeman R, Hellyer A, van der Meulen J, Thakar R. Impact of a quality improvement project to reduce the rate of obstetric anal sphincter injury: a multicentre study with a stepped-wedge design. BJOG: An Int J Obstet Gynaecol. 2021;128(3):584–92. 10.1111/1471-0528.16396 PMC7818460 33426798

[10] Bidwell P, Thakar R, Gurol-Urganci I, Harris J, Silverton L, Hellyer A (2020). Exploring clinicians’ perspectives on the “Obstetric Anal Sphincter Injury Care Bundle” national quality improvement programme: a qualitative study. BMJ Open.

[11] Bidwell P, Sevdalis N, Silverton L, Harris J, Gurol-Urganci I, Hellyer A, et al. Women’s experiences of the OASI Care Bundle; a package of care to reduce severe perineal trauma. Int Urogynecol J. 2020; In press. 10.1007/s00192-020-04653-2 PMC8295065 33475817

[12] Gillis BD, Holley SL, Parish AL. Implementation of a perinatal depression care bundle in a nurse-managed midwifery practice. Nurs Women’s Health. 2019;23(4):288–98. 10.1016/j.nwh.2019.05.007 31271731

[13] Andrews CJ, Ellwood D, Middleton PF, Homer CS, Reinebrant HE, Donnolley N, Boyle FM, Gordon A, Nicholl M, Morris J, Gardener G. Survey of Australian maternity hospitals to inform development and implementation of a stillbirth prevention ‘bundle of care’. Women Birth. 2020;33(3):251-8. 10.1016/j.wombi.2019.06.001 31227443

[14] Kawakita T, Landy HJ (2017). Surgical site infections after cesarean delivery: epidemiology, prevention and treatment. Matern Health Neonatol Perinatol.

[15] Althabe F, Therrien MNS, Pingray V, Hermida J, Gülmezoglu AM, Armbruster D, Singh N, Guha M, Garg LF, Souza JP, Smith JM, Winikoff B, Thapa K, Hébert E, Liljestrand J, Downe S, Garcia Elorrio E, Arulkumaran S, Byaruhanga EK, Lissauer DM, Oguttu M, Dumont A, Escobar MF, Fuchtner C, Lumbiganon P, Burke TF, Miller S (2020). Postpartum hemorrhage care bundles to improve adherence to guidelines: a WHO technical consultation. Int J Gynecol Obstet.

[16] Gilhooly D, Green SA, McCann C, Black N, Moonesinghe SR (2019). Barriers and facilitators to the successful development, implementation and evaluation of care bundles in acute care in hospital: a scoping review. Implement Sci.

[17] Nilsen P (2015). Making sense of implementation theories, models and frameworks. Implement Sci.

[18] Kitson A, Harvey G, Mccormack B (1998). Enabling the implementation of evidence based practice: a conceptual framework. Qual Health Care.

[19] Proctor E, Silmere H, Raghavan R, Hovmand P, Aarons G, Bunger A, Griffey R, Hensley M (2011). Outcomes for implementation research: conceptual distinctions, measurement challenges, and research agenda. Adm Policy Ment Health Ment Health Serv Res.

[20] Fernandez ME, ten Hoor GA, van Lieshout S, Rodriguez SA, Beidas RS, Parcel G, et al. Implementation mapping: using intervention mapping to develop implementation strategies. Front Public Health. 2019;7. 10.3389/fpubh.2019.00158. 10.3389/fpubh.2019.00158 PMC6592155 31275915

[21] Rycroft-Malone J, Kitson A, Harvey G, McCormack B, Seers K, Titchen A, Estabrooks C (2002). Ingredients for change: revisiting a conceptual framework. Qual Saf Health Care.

[22] Powell BJ, Waltz TJ, Chinman MJ, Damschroder LJ, Smith JL, Matthieu MM (2015). A refined compilation of implementation strategies: results from the Expert Recommendations for Implementing Change (ERIC) project. Implement Sci.

[23] NHS Improvement (2019). Implementation of evidence into practice in health and social care settings.

[24] NHS. Maternity and Neonatal Safety Improvement Programme. Available from: https://www.england.nhs.uk/mat-transformation/maternal-and-neonatal-safety-collaborative/. Accessed 30 Dec 2020.

[25] Sweeney LB, Meadows D. The systems thinking playbook: Exercises to stretch and build learning and systems thinking capabilities. Chelsea Green Publishing; 2010.

[26] Little RJA, Rubin DB. Statistical analysis with missing data. Stat Anal Missing Data. 2002. 10.1002/9781119013563.

[27] Weiner BJ, Lewis CC, Stanick C, Powell BJ, Dorsey CN, Clary AS (2017). Psychometric assessment of three newly developed implementation outcome measures. Implement Sci.

[28] Rapley T, Girling M, Mair FS, Murray E, Treweek S, McColl E, Steen IN, May CR, Finch TL. Improving the normalization of complex interventions: part 1-development of the NoMAD instrument for assessing implementation work based on normalization process theory (NPT). BMC Med Res Methodol. 2018;18(1):1–7. 10.1186/s12874-018-0590-y PMC6238361 30442093

[29] NHS Care Quality Commission (2019). 2019 Maternity survey: quality and methodology report.

[30] Harrison S, Alderdice F, Henderson J, Redshaw M, Quigley MA (2020). Trends in response rates and respondent characteristics in five National Maternity Surveys in England during 1995-2018. Arch Public Health.

[31] Boddy CR (2016). Sample size for qualitative research. Qual Mark Res.

[32] Ritchie J, Lewis J, Nicholls CM, Ormston R, editors. Qualitative research practice: A guide for social science students and researchers. Sage; 2013.

