# Supplementary figures and images for: OASI2: a cluster randomised hybrid evaluation of strategies for sustainable implementation of the Obstetric Anal Sphincter Injury Care Bundle in maternity units in Great Britain
Source: Implement Sci. 2021 May 22;16:55. doi: 10.1186/s13012-021-01125-z (PMC8140475; doi:10.1186/s13012-021-01125-z)

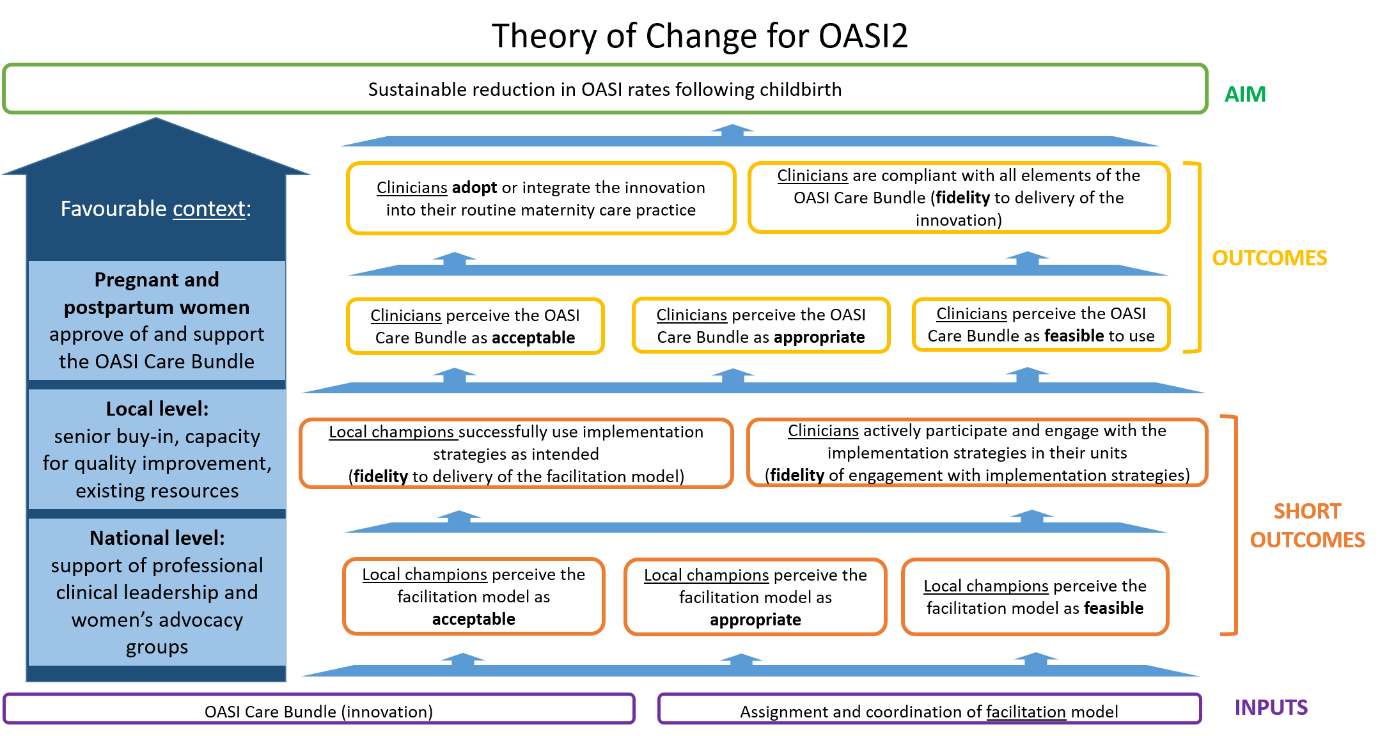
Additional File 1. Theory of Change for OASI2

Supplement: Supplementary file 1 — Additional file 1. Theory of Change for OASI2 [file 13012_2021_1125_MOESM1_ESM.docx]
