# Supplementary material for: OASI2: a cluster randomised hybrid evaluation of strategies for sustainable implementation of the Obstetric Anal Sphincter Injury Care Bundle in maternity units in Great Britain
Source: Implement Sci. 2021 May 22;16:55. doi: 10.1186/s13012-021-01125-z (PMC8140475; doi:10.1186/s13012-021-01125-z)
Supplement: Supplementary file 2 — Additional file 2. Summary of data specification for the OASI2 project [file 13012_2021_1125_MOESM2_ESM.docx]

Additional file 2. Summary of data specification for the OASI2 project

- This is a summary of the data items that will be requested all births**.** All information requested is essential for both linkage and risk-adjustment. If data is limited, simply send what is available but be sure that data items marked with a double-asterisk (**) are included at minimum.
- No personal data (e.g. mother’s name or address) will be requested—pseudonymised identifiers must be generated at the site level, ensuring that data is non-identifiable by the Project Team but traceable by the Trust in order to respond to any data quality/coding-related queries.
- The ‘OASI Care Bundle use’ variable (in bold below) is the only item that is not routinely collected. We will work with each site to determine the best way to collect this data and include it in the quarterly data transfers—it is not mandatory for the baseline extract for the year prior to OASI2 start.

|  | Data item | (Data item name based on MSDS  XML schema where possible) |
| --- | --- | --- |
| Demographic | **Mother’s pseudonymised ID | PseudonymisedID |
|  | **Mother’s age [risk factor] | AgeAtBookingMother |
|  | **Mother’s ethnicity [risk factor] | EthnicCategoryMother |
|  | Mother’s first language (Mother at booking) | FirstLanguageEnglishIndMother |
| Obstetric history | **Parity [risk factor] | PrevTotalBirths |
|  | Number of previous caesarean births | PreviousCaesareanSections |
|  | Number of previous instrumental births | PreviousInstrumentalBirths |
|  | Number of previous third or fourth degree perineal tears | PreviousSeverePerinealTrauma |
| Antenatal care | Maternal weight at booking | MotherWeight |
|  | Maternal height | MotherHeight |
|  | **BMI at booking | MotherBMI |
|  | Maternal obstetric diagnosis type (current pregnancy) | PregnancyObstetricDiagType |
|  | Maternity complicating medical diagnosis (mother at booking) | ComplicatingDiagTypeMother |
| Labour and Delivery | **Site code of actual place of delivery | SiteCodeActualDelivery |
|  | **Actual place of birth category | PlaceTypeActualDelivery |
|  | Delivery place actual midwifery unit type | PlaceTypeActualMidwifery |
|  | Number of infants this delivery | NumberInfants |
|  | Onset of labour | LabourOnset |
|  | Pain relief type (labour and delivery) | LabourPainReliefMethod |
|  | Anaesthesia in labour and delivery | LabourAnaesthesiaType |
|  | Status of clinician who attended birth | ClinicianAtDelivery |
|  | Clinicians supervised by (if applicable) | SeniorPersonAtDelivery |
|  | **Presentation at delivery | DeliveryPresentation |
|  | **Method of delivery | DeliveryMethodBaby |
|  | **Delivered in water | WaterDeliveryInd |
|  | **Genital tract trauma [outcome—including vaginal wall, labial tears, degree of perineal tear] | GenitalTractTraumaticLesion |
|  | **Episiotomy [indication & outcome] | Episiotomy |
|  | **OASI-Care Bundle Use [were all four components of the OASI-CB used in this birth?]** |  |
| Baby | **Date of birth of delivery [In MM/YYYY format] | BabyBirthMonth |
|  | **Delivery outcome | FetusOutcome |
|  | **Birth weight [risk factor] | BirthWeight |
|  | **Gestational age at birth in days | GestationLengthBirth |
|  | **Baby complications at birth [shoulder dystocia--risk factor] | BabyComplicationAtBirth |
